# „Sterbehilfe und Suizid“ – Ein Fallbericht über den begleiteten Suizid im Rahmen psychischer Erkrankungen

**DOI:** 10.1007/s40211-025-00523-9

**Published:** 2025-03-19

**Authors:** Cornelia Marion Diendorfer, Dominik Ivkic, Valentin Popper, Matthäus Willeit, Christoph Kraus

**Affiliations:** Universitätsklinik für Psychiatrie und Psychotherapie, Klinische Abteilung für Allgemeine Psychiatrie, AKH, Medizinische Universität Wien, Währinger Gürtel 18–20, 1090 Wien, Österreich

**Keywords:** Assistierter Suizid, Fallbericht, Psychische Erkrankungen, Gesetzgebung, Medizinethik, Assisted suicide, Case report, Mental illnesses, Legislation, Medical ethics

## Abstract

Seit Januar 2022 ist auch in Österreich die Beihilfe zum Suizid unter bestimmten rechtlich festgelegten Voraussetzungen erlaubt. Laut Verfassungsgerichtshof entsprach die frühere Gesetzgebung nicht dem „Recht auf Selbstbestimmung“ und wurde als verfassungswidrig erklärt [1]. Im Vergleich dazu haben Länder wie beispielsweise die Niederlande, Belgien und die Schweiz bereits seit längerem eine weniger restriktive Gesetzgebung zur Sterbehilfe. Voraussetzung für die Einreichung eines Antrags auf assistierten Suizid ist, dass der Patient/die Patientin an einer unheilbaren Erkrankung leidet und sowohl die Entscheidungsfähigkeit als auch der freie Wille der Person sichergestellt sind [1]. Der vorliegende Fallbericht einer 47-jährigen Patientin, die an einer rezidivierenden depressiven Störung mit gegenwärtig schwerer Episode litt und sich nach zwei Suizidversuchen in der Vergangenheit für einen assistierten Suizid in der Schweiz entschied, soll die ethischen und rechtlichen Herausforderungen beleuchten, die mit der Beihilfe zum Suizid für psychiatrische PatientInnen verbunden sind, und die Notwendigkeit einer sorgfältigen Abwägung zwischen Autonomie und Schutzpflicht betonen.

## Abstract (en)

Since January 2022, assisted suicide has also been legal in Austria under certain legally specified conditions. According to the Constitutional Court, the previous legislation did not align with the “right to self-determination” and was therefore declared unconstitutional [1]. In contrast, countries such as the Netherlands, Belgium, and Switzerland have had a less restrictive legislation on assisted suicide for a longer period of time. The requirement for submitting a request for assisted suicide is that the patient suffers from a terminal illness and that both their decision-making capacity and free will are ensured [1]. This case report of a 47-year-old patient, who suffered from recurrent depressive disorder with a current severe episode and, after two previous suicide attempts, chose assisted suicide in Switzerland, aims to highlight the ethical and legal challenges associated with assisted suicide for psychiatric patients and to emphasize the need for careful consideration between autonomy and the duty of protection.

## Einleitung

Seit Januar 2022 ist die Beihilfe zum Suizid in Österreich unter bestimmten Voraussetzungen nicht mehr strafbar. Dies geht auf ein Urteil des Verfassungsgerichtshofs (VfGH) zurück, der die frühere Gesetzgebung als verfassungswidrig einstufte, da sie das „Recht auf Selbstbestimmung“ nicht ausreichend gewährleistete. Seitdem haben PatientInnen auch in Österreich erstmals die Möglichkeit, eine sogenannte Sterbeverfügung zu errichten [1]. Im Gegensatz zur „aktiven Sterbehilfe“ bzw. „Euthanasie“, bei der beispielsweise durch medizinisches Personal ein tödlich wirkendes Medikament unmittelbar verabreicht wird, erfolgt beim assistierten Suizid lediglich die Bereitstellung des Medikaments, die Einnahme muss jedoch durch den Patient/die Patientin eigenständig erfolgen [2–4]. Die Begriffe „Euthanasie“ und „Sterbehilfe“ werden in der Literatur oft synonym verwendet (siehe Tab. 1). Allerdings wird der Ausdruck „Euthanasie“ im deutschsprachigen Raum aufgrund der belastenden historischen Assoziationen mit den Euthanasie-Programmen des Dritten Reichs weitgehend vermieden [5]. Während in Ländern wie den Niederlanden oder Belgien sowohl die aktive Sterbehilfe als auch der assistierte Suizid bereits seit längerem unter bestimmten Umständen legal sind [5], ist die „aktive Sterbehilfe“ in Österreich weiterhin verboten.

**Tab. 1:** Formen der Sterbehilfe

*Aktive Sterbehilfe*
Beenden des Lebens eines anderen Menschen auf dessen Wunsch, z. B.: durch eine Medikamentenüberdosis
*Indirekte Sterbehilfe*
Nicht primär intendierte Beschleunigung des Todeseintritts, z. B.: als Nebenwirkung durch Verabreichung schmerzlindernder Medikamente
*Passive Sterbehilfe*
Unterlassen von lebensverlängernden Maßnahmen, z. B.: Abschalten eines Beatmungsgeräts
*Assistierter Suizid*
Selbsttötung mit Hilfe einer Person, die ein Mittel hierfür bereitstellt; Mittel muss von erkrankter Person eigenständig eingenommen werden

Um in Österreich eine Sterbeverfügung für einen assistierten Suizid errichten zu können, ist eine ärztliche Aufklärung erforderlich. Diese muss durch zwei ÄrztInnen erfolgen, von denen einer/eine über eine Qualifikation in Palliativmedizin verfügen muss.

Diese Aufklärung umfasst Informationen über die Diagnose, den Krankheitsverlauf, palliative Alternativen und die Durchführung des assistierten Suizids. Des Weiteren müssen PatientInnen zum Zeitpunkt der Errichtung der Sterbeverfügung entscheidungsfähig sein, das heißt, sie müssen die Bedeutung und die Folgen ihrer Entscheidung verstehen und bewerten können. Eine weitere Voraussetzung ist das Vorliegen eines Leidenszustands: PatientInnen müssen an einer unheilbaren, zum Tode führenden oder an einer schweren, chronischen Krankheit mit anhaltenden Symptomen leiden, die die Lebensqualität dauerhaft beeinträchtigen. Zwischen der ersten ärztlichen Aufklärung und der tatsächlichen Errichtung der Sterbeverfügung muss eine Wartefrist von mindestens zwölf Wochen eingehalten werden; bei PatientInnen mit einer terminalen Erkrankung kann diese Frist auf zwei Wochen reduziert werden. Die Sterbeverfügung muss schriftlich bei PatientenanwältInnen oder NotarInnen errichtet und schließlich in einem zentralen Register registriert werden. Diese rechtlichen Änderungen und ihre praktischen Implikationen sind von großer Bedeutung für die medizinische Praxis und die Rechte der PatientInnen in Österreich [1].

Im Vergleich dazu sind die Gesetze zur Sterbehilfe in Ländern wie den Niederlanden, Belgien oder der Schweiz bereits seit vielen Jahren etabliert und weniger restriktiv. Die Möglichkeit, einen assistierten Suizid auch ohne das Vorliegen einer terminalen Erkrankung in Anspruch nehmen zu können, wird durch die Tatsache ermöglicht, dass unerträgliches Leiden und das Fehlen weiterer verfügbarer Behandlungsoptionen ausreichen, um einen assistierten Suizid zu rechtfertigen [6]. Regelungen wie diese ermöglichen es, Menschen mit psychischen Erkrankungen, deren Symptom Suizidalität ist, einen assistierten Suizid wählen zu können.

In der Schweiz haben sich mittlerweile verschiedene Organisationen etabliert, die Menschen unterstützen, die sich für einen begleiteten Suizid entscheiden. Diese Organisationen setzen eine bestehende Urteilsfähigkeit und dass die Entscheidung für einen assistierten Suizid aus freiem Willen erfolgt voraus. Im Gegensatz zu den Bestimmungen in Österreich ist jedoch keine ärztliche Begutachtung durch zwei unterschiedliche ÄrztInnen erforderlich, von denen einer/eine eine palliativmedizinische Ausbildung haben muss. Bei den genannten Organisationen in der Schweiz wird die Krankengeschichte sowie ärztliche Befunde an einen kooperierenden Arzt übermittelt, der das Rezept für das letale Medikament ausstellt. Die Inanspruchnahme des Angebots dieser Organisationen ist in der Regel kostenpflichtig und die Kriterien für die Zustimmung zum assistierten Suizid können variieren. Einige Organisationen bieten zudem auch Menschen aus anderen Ländern die Möglichkeit, legal in der Schweiz einen assistierten Suizid in Anspruch nehmen zu können [7].

## Fallbericht

Eine 47-jährige Patientin wurde zur Evaluation einer möglichen Spravato-Therapie von einem niedergelassenen Facharzt für Psychiatrie an die psychiatrische Ambulanz der Medizinischen Universität Wien überwiesen. Bei der Patientin war zu diesem Zeitpunkt die Diagnose einer rezidivierenden depressiven Störung bekannt und sie hatte den Entschluss gefasst, einen assistierten Suizid in Anspruch zu nehmen. Aufgrund der Annahme, dass in Österreich die Bewilligung durch zwei unterschiedliche ÄrztInnen erforderlich wäre, und die damit verbundenen Auflagen möglicherweise strenger und der Prozess langwieriger sein könnten, hatte sich die Patientin entschieden, einen assistierten Suizid über eine Organisation in der Schweiz in Anspruch zu nehmen und die logistischen Schritte bereits eingeleitet. Die Vorstellung beim niedergelassenen Psychiater erfolgte, da die Patientin von diesem eine Bestätigung zur Bewilligung des assistierten Suizids wünschte. Jener Psychiater stellte im Rahmen der Exploration fest, dass bei der Patientin in der Vergangenheit, entgegen der gegenwärtigen Leitlinien für die Behandlung einer therapieresistenten Depression [8], weder eine Ketamin‑/Esketamin-Therapie noch eine Elektrokonvulsionstherapie (EKT) durchgeführt wurden. Er überwies die Patientin daher an unsere Ambulanz zur erstmaligen Behandlung mit Esketamin i. n.

Es gab in der Vergangenheit der Patientin mehrere Therapieversuche mit diversen Antidepressiva, inklusive Venlafaxin, Mirtazapin, Sertralin, Escitalopram, Bupropion, Citalopram, Agomelatin, Trazodon, Duloxetin, sowie einen Augmentationsversuch mit Risperidon.

Zum Zeitpunkt des ersten Ambulanzkontaktes an unserer Klinik nahm die Patientin als Dauermedikation Mirtazapin 30 mg Tagesdosis (TD), Venlafaxin 150 mg TD und Pregabalin 100 mg TD ein. Im Rahmen einer Laboruntersuchung der jeweiligen Medikamentenspiegel im Blut zeigten sich die folgenden Werte: Mirtazapin 46,6 ng/mL (TB: 30–80 ng/mL), Venlafaxin 34,3 ng/mL (TB: 30–180 ng/mL) und O‑Desmethylvenlafaxin 237,7 (TB: 100–400 ng/mL).

Hinsichtlich früherer psychotherapeutischer Behandlungen gab die Patientin an, vor etwa 10 Jahren über einen Zeitraum von zwei Jahren regelmäßig an einer ambulanten Psychotherapie teilgenommen zu haben. Sie konnte jedoch nicht angeben, welche spezifische Therapieform angewendet wurde. Sie berichtete, dass sie keinerlei Besserung durch die Psychotherapie wahrgenommen und die Behandlung daher im weiteren Verlauf abgebrochen habe.

Die Patientin erhielt in weiterer Folge an der Wiener Universitätsklinik für Psychiatrie und Psychotherapie eine Esketamin-Therapie i. n., bestehend aus insgesamt acht Verabreichungen. Aufgrund einer unzureichenden Response wurde schließlich eine stationäre Aufnahme zur weiteren Behandlung vereinbart und die Durchführung einer erstmaligen EKT-Serie in Betracht gezogen.

### Biografische Daten

Die Patientin wuchs als ältestes von insgesamt vier Kindern auf. Laut eigener Angaben habe sie bereits in der Kindheit kaum Freundschaften gehabt und generell sozial sehr zurückgezogen gelebt. Sie berichtete von sowohl psychischer als auch physischer Gewalt während ihrer Kindheit, welche bereits früh ein Gefühl der Macht- und Hilflosigkeit in ihr ausgelöst habe. Bereits im Alter von vier Jahren habe sie erstmals depressive Symptome bei sich wahrgenommen. Die Patientin berichtete insgesamt von mindestens drei depressiven Episoden in der Vergangenheit in Form eines rezidivierenden Verlaufs ohne Remission. In ihrem Leben habe sie lediglich einmalig eine halbjährige „gute Phase“ vor etwa 20 Jahren gehabt. Damals seien eine funktionierende Beziehung und ein erfüllender Job vorhanden gewesen. Seither habe die Patientin jedoch nicht mehr in einer Partnerschaft gelebt. Familiären Kontakt habe sie vor allem zu ihrer jüngeren Schwester gepflegt, ansonsten war das Verhältnis zu ihrer Familie eher ablehnend. Sie gab an, dass die zum Zeitpunkt der Aufnahme vorhandene depressive Symptomatik bereits seit über 2 Jahren durchgehend bestanden habe. Nicht suizidales selbstverletzendes Verhalten in der Vergangenheit negierte die Patientin. Insgesamt habe es in der Vergangenheit der Patientin zwei Suizidversuche gegeben. Diese lagen etwa 10 Jahre zurück. Beide seien in Form einer Intoxikation mittels einer Kombination aus Zoldem und Alkohol erfolgt. Außerdem fanden sich in der Vergangenheit mehrere psychiatrisch-stationäre Voraufenthalte. 2022 kam es schließlich zu einer akuten Aggravierung der depressiven Symptomatik aufgrund von vermehrtem Stress im Berufsleben.

### Psychopathologischer Status bei Aufnahme

Zum Zeitpunkt der Aufnahme zeigte sich die Patientin bewusstseinsklar, allseits voll orientiert. Auffassung, Konzentration und Mnestik waren grobklinisch unauffällig. Der Gedankenductus war im Tempo diskret beschleunigt, aber kohärent und zielführend. Es war keine produktiv-psychotische Symptomatik explorierbar. Laut Patientin habe eine generalisierte Angst bestanden. Keine Zwangsgedanken oder -handlungen. Die Patientin war weinerlich, Affizierbarkeit in beiden Skalenbereichen erschwert, aber möglich. Antrieb war subjektiv reduziert. Psychomotorik diskret gesteigert. Einschlaf- und Durchschlafstörungen vorhanden. Appetit unauffällig. Chronische Suizidgedanken vorhanden und Wunsch nach assistiertem Suizid in der Schweiz. Zum Zeitpunkt der Exploration waren keine unmittelbare Selbst- oder Fremdgefährdung fassbar.

- *Somatischer Status: *unauffällig
- *Neurologischer Status: *unauffällig
- *Somatische Vorerkrankungen*: Hashimoto Thyreoiditis

### Diagnostik

Die Diagnostik wurde mittels ICD-10 und DSM-5 Kriterien durchgeführt. Zur störungsspezifischen Diagnostik wurden zudem die „Montgomery Asberg Depression Rating Scale“ (MADRS) und „AQ 50 – Autism Spectrum Quotient“ angewendet.

### Stationärer Aufenthalt und Behandlung

**Abb. 1:**
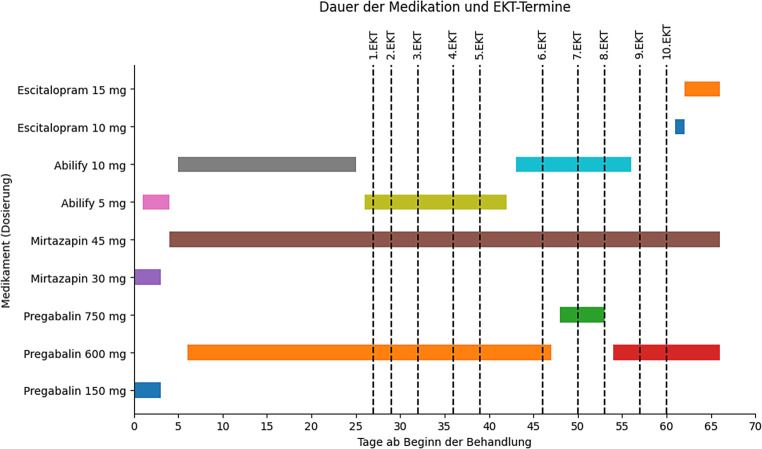
Zeitlicher Verlauf der Medikamentenänderungen und EKT-Behandlungen ab stationärer Aufnahme

Zum Zeitpunkt der stationären Aufnahme bestand als Dauermedikation Mirtazapin 30 mg TD und Pregabalin 150 mg TD. Das voretablierte Venlafaxin war von der Patientin inzwischen selbstständig abgesetzt worden. Aufgrund der starken inneren Unruhe und Ängstlichkeit der Patientin wurde Pregabalin schrittweise auf insgesamt 600 mg TD gesteigert. Als zusätzliche antidepressive Augmentationstherapie wurde Aripiprazol begonnen und auf 10 mg TD gesteigert. Zusätzlich wurde als antidepressive Therapie und zur Behandlung der Ängstlichkeit Escitalopram etabliert und auf 15 mg TD erhöht. Die Patientin berichtete darunter von einer Reduktion ihres ängstlichen Grübelns. Neben der Adaption der psychopharmakologischen Medikation erfolgten zudem im Rahmen des Aufenthalts regelmäßig psychotherapeutische Einzelgespräche (siehe Abb. 1).

**Abb. 2:**
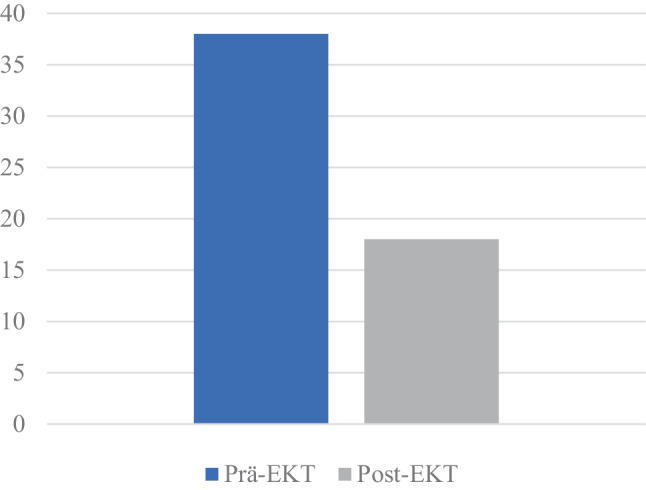
MADRS Gesamtpunktezahl der Patientin Prä- und Post-EKT

Die Patientin willigte schließlich im weiteren Verlauf neben der psychopharmakologischen Umstellung und den psychotherapeutischen Gesprächen in eine erstmalige Elektrokonvulsionstherapie ein und erhielt insgesamt 10 Behandlungen. Nach Beendigung der Serie zeigte ein erneut durchgeführter MADRS eine Reduktion des Gesamtwerts auf 18 Punkte im Vergleich zum Aufnahmetag (38 Punkte) (siehe Abb. 2).

**Abb. 3:**
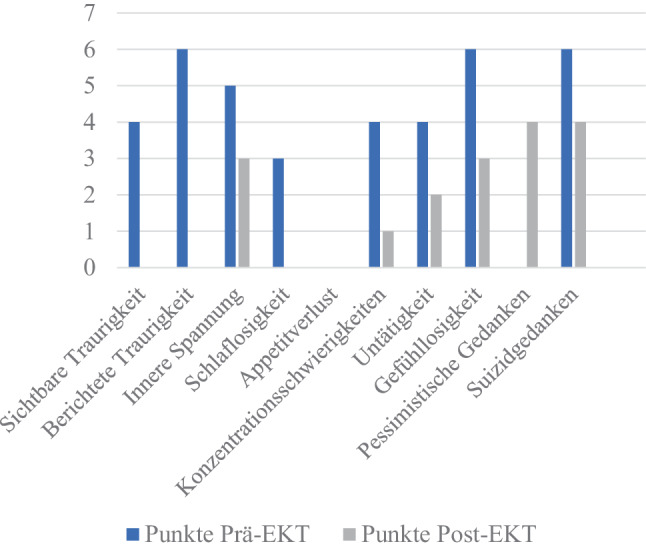
Erreichte Punkte der Patientin bei den einzelnen Items des MADRS Prä- und Post-EKT

Insbesondere die Items „Innere Anspannung“, „Konzentrationsschwierigkeiten“, „Untätigkeit“, „Gefühlslosigkeit“, „Pessimistische“- und „Suizidgedanken“ zeigten einen Rückgang (siehe Abb. 3). Insgesamt wurde bei der Patientin auch innerhalb des Teams eine reduzierte Anspannung und verbesserte Affizierbarkeit wahrgenommen, dennoch blieb der Sterbenswunsch der Patientin unverändert erhalten.

Im Rahmen des stationären Aufenthalts erfolgte des Weiteren eine klinisch psychologische Testung. Diese zeigte eine überdurchschnittlich gute intellektuelle Leistungsfähigkeit, keinerlei kognitive Beeinträchtigungen, eine unauffällige allgemeine unspezifische Leistungsfunktion, sowie einen unauffälligen Gedächtnisbefund. State- und Trait-Angst waren jedoch stark erhöht. Es waren zudem hypochondrische Tendenzen fassbar und es fanden sich schizoid vermeidend-unsichere und zwanghafte Persönlichkeitsanteile. Im AQ 50-Autism Spectrum Quotient erreichte die Patientin 32 Punkte, was mit einer 80 % Wahrscheinlichkeit für eine Autismus-Spektrum-Störung spricht. Zum Ausschluss etwaiger zugrunde liegender organischer Ursachen erfolgte zudem eine Magnetresonanztomographie (MRT), die einen unauffälligen Befund zeigte.

Während des stationären Aufenthalts kam es zu mehreren ausführlichen explorativen und diagnostischen Gesprächen. In diesen erzählte die Patientin unter anderem über ihre traumatisierende Kindheit, während der sie psychische und physische Misshandlungen erlitten habe und gab an, über dies bisher noch niemals mit jemanden gesprochen zu haben. In weiterer Folge wurde im Rahmen der psychotherapeutischen Gespräche auch Psychoedukation zum Thema Trauma geführt und versucht, die Patientin zu einer weiterführenden Traumatherapie zu motivieren. Dies lehnte die Patientin jedoch mit der Begründung ab, sich bereits für einen assistierten Suizid entschieden zu haben.

Diagnostisch lag bei der Patientin am ehesten eine ängstlich-agitierte depressive Symptomatik vor. Die Patientin erfüllte die Kriterien einer posttraumatischen Belastungsstörung (PTBS: Schlafstörungen, vegetatives Hyperarousal u. Schreckhaftigkeit, vermehrte Angst) mit andauernder Persönlichkeitsänderung (F62.0). Sie zeigte hierbei jedoch zahlreiche Ressourcen, war kommunikativ, konnte komplexe Informationen rasch verarbeiten und zeigte sich im Rahmen des stationären Settings stets verlässlich. Auffallend waren jedoch ihre wiederholt geäußerten Sorgen, sie würde mit ihrem Krankenhausaufenthalt und der Therapie dem Gesundheitssystem zur Last fallen oder anderen PatientInnen, die ihrer Ansicht nach dringlicher Hilfe benötigten, den Platz wegnehmen. Zudem äußerte sie wiederholt in einem inneren Konflikt zu stehen, inwieweit sie ihre Familie hinsichtlich ihres Vorhabens informieren solle.

### Weiterer Verlauf

Nach Beendigung der EKT-Serie entließ sich die Patientin gegen ärztlichen Rat schließlich selbst. Trotz wiederholter Versuche die Patientin an eine ambulante Psychotherapie zu vermitteln, lehnte sie dies beharrlich ab. Sie wurde schließlich von unserer Seite hinsichtlich der Vorstellung ihres Falles im Rahmen einer klinikinternen Fallkonferenz kontaktiert und erklärte sich nicht nur dazu bereit, dass dieser besprochen werden dürfe, sondern sie auch persönlich daran teilnehmen wolle. So folgten vorab zunächst mehrere weitere Ambulanzkontakte. Darin berichtete die Patientin ihre verordnete psychopharmakologische Medikation unmittelbar nach der stationären Entlassung zur Gänze abgesetzt zu haben. Als Motivationsgrund für ihre Teilnahme an der Fallkonferenz gab sie an, dass sie gerne alles dafür tun wolle, um noch einen wissenschaftlichen Beitrag leisten zu können und unterzeichnete eine Einverständniserklärung zur Publikation ihres Fallberichts. So erklärte sie sich in weiterer Folge auch bereit, einen off-label Therapieversuch mit Clozapin zu starten, für welches in der Literatur eine antisuizidale Wirksamkeit beschrieben ist [9]. Es erfolgte eine Dosissteigerung auf insgesamt 100 mg TD. Die weitere Therapie wurde jedoch von der Patientin nach nur wenigen Wochen wieder beendet und sie benachrichtigte uns per E‑Mail, dass sie den assistierten Suizid wie geplant in der Schweiz in Anspruch nehmen werde. Die Patientin verstarb zwei Monate nach dem letzten ambulanten Kontakt in der Schweiz.

## Diskussion

Der assistierte Suizid in Österreich bei psychiatrischen PatientInnen stellt ein ethisch und rechtlich komplexes und kontroverses Thema dar. Der hier beschriebene Fallbericht soll diese Komplexität näher veranschaulichen. Zum Zeitpunkt des in der Schweiz eingeleiteten Verfahrens für einen assistierten Suizid waren bei der Patientin noch nicht sämtliche therapeutische Maßnahmen gemäß den aktuellen Leitlinien ausgeschöpft. Insbesondere für Ketamin- und EKT-Behandlungen wird in der Fachliteratur eine schnell einsetzende antisuizidale Wirkung beschrieben [8]. Zudem offenbarte die Patientin während ihres stationären Aufenthalts erstmals schwerwiegende Traumafolgen, für die sie bislang keine spezifische Traumatherapie erhalten und insgesamt keine adäquate längerfristige psychotherapeutische Behandlung erfahren hatte. Es zeigte sich jedoch, dass die Patientin grundsätzlich kooperativ in der Psychotherapie war und nach einigen Sitzungen positive Effekte spürte. Vor diesem Hintergrund konnte man erwarten, dass eine kontinuierliche psychotherapeutische Behandlung potenziell vorteilhafte Auswirkungen auf ihren weiteren Krankheitsverlauf gehabt hätte. Die Zusage eines assistierten Suizids in der Schweiz trotz solcher Behandlungslücken löst erhebliche Bedenken aus. Voraussetzung für einen assistierten Suizid ist, dass PatientInnen entweder an einer unheilbaren, zum Tod führenden oder an einer schweren, chronischen Krankheit leiden, die die Lebensqualität dauerhaft beeinträchtigt. Die Tatsache, dass auch unerträgliches Leiden als Voraussetzung ausreichend ist, ermöglicht es somit Menschen mit psychischen Erkrankungen unter Umständen einen begleiteten Suizid in Anspruch zu nehmen. Studien zeigen, dass viele Menschen mit schweren psychischen Erkrankungen, wie chronischer Depression oder Schizophrenie, ihre Lebensqualität als unerträglich empfinden und wiederholte Suizidversuche unternehmen [10, 11].

Es ergibt sich hier jedoch die Schwierigkeit der Definition von „unerträglichem Leid“, da es sich hierbei um ein subjektives Empfinden handelt und dieses nicht objektiviert werden kann. Somit könnten sich hier die Einschätzungen je nach Behandler deutlich unterscheiden [11]. Zudem ist zu berücksichtigen, dass besonders im Rahmen von Persönlichkeitsstörungen häufiger eine intensivere Auseinandersetzung mit Suizidgedanken und -plänen beobachtbar ist [5]. In einer niederländischen Analyse der zwischen 2011–2014 durchgeführten assistierten Suizide zeigte sich, dass innerhalb jener Gruppe, die aufgrund einer psychiatrischen Diagnose einen begleiteten Suizid wählten, 52 % der Betroffenen an einer Persönlichkeitsstörung litten. Zudem zeigte sich eine höhere Prävalenz einer Persönlichkeitsstörung bei jüngeren PatientInnen [12]. Insbesondere bei Persönlichkeitsstörungen zeigen Suizidgedanken und -pläne allerdings häufig einen fluktuierenden Verlauf und sind unter Umständen durch eine angemessene Therapie suffizient behandelbar. Dies deutet darauf hin, dass es sinnvoll sein könnte, eine sorgfältige psychiatrische Diagnosestellung im Kontext einer Sterbeverfügung in Betracht zu ziehen, um sicherzustellen, dass alle therapeutischen Optionen ausgeschöpft und die Entscheidungsfähigkeit der Betroffenen umfassend bewertet wird.

**Abb. 4:**
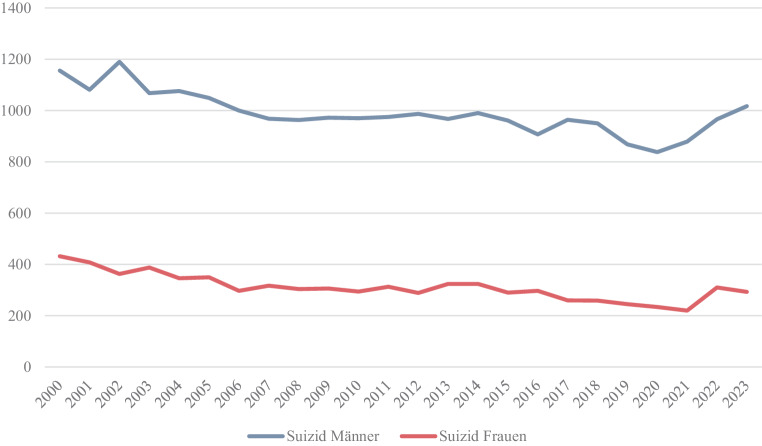
Anzahl der Suizide in Österreich in absoluten Zahlen [15]

**Abb. 5:**
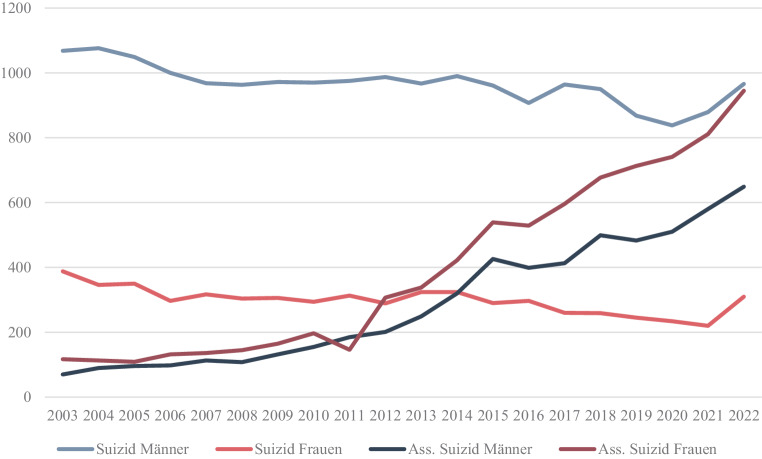
Suizide und assistierte Suizide in Schweiz in absoluten Zahlen aufgeteilt hinsichtlich Geschlecht [16–18]

Seit der Legalisierung des assistierten Suizids in Ländern wie der Schweiz, Belgien und der Niederlande konnte sowohl ein allgemeiner als auch speziell in der Gruppe der psychisch Erkrankten, stetiger Anstieg von entsprechenden Anfragen verzeichnet werden [13]. Die Anzahl herkömmlicher Suizide blieb dagegen jedoch über die vergangenen Jahre nahezu konstant (siehe Abb. 4 und 5). Unter den Schweizer Staatsbürgern, die einen assistierten Suizid wählten, litten 2006 8 % an einer psychischen Erkrankung. Unter den Antragsstellern aus anderen Herkunftsländern waren es sogar 17 % der Fälle [14, 11]. Allgemein dürften sich tendenziell mehr Frauen für einen assistierten Suizid entscheiden (siehe Abb. 5). Dies ist insofern interessant, da grundsätzlich herkömmliche Suizide häufiger von Männern begangen werden (siehe Abb. 4 und 5).

Der Zugang zum assistierten Suizid stellt die Autonomie des Einzelnen in Konflikt mit der Pflicht der MedizinerInnen und der Gesellschaft, besonders vulnerable Gruppen zu schützen. Psychiatrische PatientInnen sind häufig besonders verletzlich und können in ihren Urteils- und Entscheidungsfähigkeiten eingeschränkt sein. Die Diagnose einer psychischen Erkrankung wirft die Frage auf, ob der Wunsch nach Suizid tatsächlich Ausdruck eines autonomen Willens oder nicht vielmehr ein Symptom der Erkrankung ist [19]. Psychiatrische Erkrankungen sind oft episodisch und behandelbar, sodass der Suizidwunsch in akuten Phasen auftreten kann, aber nicht von Dauer sein muss [11].

Die Differenzierung zwischen einem wohlüberlegten Suizidwunsch, der frei von krankheitsbedingten Einflüssen ist, und einem durch eine psychische Erkrankung geprägten Drang stellt eine erhebliche Herausforderung dar. PsychiaterInnen müssen beurteilen, ob ein Patient/eine Patientin in der Lage ist, die Konsequenzen seines/ihres Handelns vollständig zu verstehen und abzuschätzen und es sollte in Erwägung gezogen werden, diese in den Entscheidungsprozess zu involvieren.

Der Anstieg der Inanspruchnahme eines assistierten Suizids unter psychisch Erkrankten verdeutlicht die Notwendigkeit, sorgfältige Kriterien festzulegen, insbesondere in Bezug auf die Entscheidungsfähigkeit der Betroffenen. Das Fehlen einer psychiatrischen Begutachtung in dem Prozess kann jedoch dazu führen, dass psychische Erkrankungen mit dem gegebenenfalls behandelbaren Symptom Suizidalität nicht ausreichend erkannt werden könnten. Eine sorgfältige und individualisierte Prüfung jedes Falls, umfassende psychologische und psychiatrische Begutachtungen von hoher Qualität und Therapien sind unerlässlich, um medizinisch richtige Entscheidungen zu treffen.

Dieser Fallbericht soll die Vielschichtigkeit dieses komplexen und kontroversen Themas veranschaulichen und zu einer vertieften Auseinandersetzung anregen, da Gesellschaft und Medizin künftig zunehmend mit der Herausforderung konfrontiert sein werden, solche Fälle verantwortungsvoll zu bewerten und zu begleiten.
